# Supplementary material for: Correlation between clinical disease activity and interferon-γ autoantibody titers measured by inhibitory ELISA, and inflammatory biomarkers in adult-onset immunodeficiency associated with anti-interferon-γ autoantibodies
Source: PLoS One. 2026 Mar 18;21(3):e0344248. doi: 10.1371/journal.pone.0344248 (PMC12998871; doi:10.1371/journal.pone.0344248)
Supplement: Attachment — Submitted filename: Manuscript PONE 025 58456 dec 2025.pdf [file pone.0344248.s001.pdf]

Manuscript PONE-D-25-58456, entitled "Correlation between clinical disease activity and interferon-Y autoantibody titers measured by inhibitory ELISA, and inflammatory biomarkers in adult-onset immunodeficiency associated with anti-interferon-Y autoantibodies".

MANUSCRIPT CRITERIA FOR REVIEW:

Anti-IFN-γ AAbs and active/remission disease in NTM infections

1. The study presents the results of original research.

Yes. The study is an original research not done previously. Manuscript analysis of clinical and laboratory data is well designed. Available laboratory data for diagnosis of immunodeficiency are appropriate to measure disease activity due to NTM infections. Differences were significant to differentiate between active and remission disease not only for a-IFNg Ab but also for the rest of inflammatory markers. But the study doesn´t mention issues like duration of treatment, doses, duration time of remission, relapses, comodulatory IS treatments and so. There isn´t a follow-up schema of development. It is something like a cut-off for each clinical step condition.

2. Results reported have not been published elsewhere.

No. Similar results haven´t been published yet, nor if similar treatments had been done. There are only 26 reference papers regarding the identification of a-IFNg AAb. Most of studies in similar treatment cases are refered to use of immunomodulators or corticoid therapy not just as anti-aIFNg AAbs

3. Experiments, statistics, and other analyses are performed to a high technical standard and are described in sufficient detail.

Yes, but description of material and methods are brief as simple timepoints as active or in remission following therapy. But it doesn´t mention relapse nor time of follow-up. In my opinión details aren´t enough descriptive.

4. Conclusions are presented in an appropriate fashion and are supported by the data.

Yes, conclusions are descriptive as they are reported in the manuscript but show very scarce data. There aren´t previous data of development of disease nor which ones AAbs are related to what kind of morbidity disease is more prone to development of AAbs or how much time sparsed after adult-onset immunodeficiency was diagnosed.

5. The article is presented in an intelligible fashion and is written in standard English.

Yes, the article is intelligible and written in standard English.

6. The research meets all applicable standards for the ethics of experimentation and research integrity.

Yes. The article meets standard measures for ethics of experimentations, but these are briefly reported and aren´t enough descriptive. Previous comorbidity pathologic conditions doesn´t mention in terms of laboratory data if earliest disease manifestations can be related to the so called initial adult-onset immunodeficiency disease not to what this rising would be due, nor if there are previous treatments achieved during early comorbidities, evolution or manifestations of disease to best define initial adult-onset criteria for diagnosis.

The analysis for perfomance of a-IFNg Ab doesn´t mention any explicative change regarding the drop of a-IFNg Ab. Manuscript mentions there aren´t laboratory changes regarding population of immunocompetent T, B and NK cells between early clinical diagnosis and following remission. On the other hand, analysis doesn´t mention changes for expresión of activation markers among immunocompetent population nor if there is a shift from an inflammatory Th1 profile to another anti-inflammatory Th2 population, or viceversa and even in the secretion of cytokine profiles of subpopulations. It would be appropriate to know if such immunologic adult-onset disease parameters are related to initial clinical course changes or from active to remission of disease, following a-IFNg AAbs changes. There is also a lack of other inflammatory and/or fibrin- or coagulation-related factors, such as ferritin, D-dimer, PLT and thrombin-related times which would perpetuate inflammatory profiles. These alterations can condition initial antiinflammatory response to achieve remission or avoiding it or deterioration due to previous pathologic unknow alterations.

Most of studied patients seem to respond to treatments but there aren´t reports of failure, due to such immunomodulatory therapy. In my opinión, there are some issues that remain to be defined in a context-dependent manner as in maintaining active treatment in order to avoid progression and to what extent patients remain free of disease after ending treatment.

In summary changes in remission states have their own positive clinical characteristics at remission contrary to those at initial adult-onset diagnosis but it means to check clinical course of disease along time in order to maintain anti-inflammatory changes.

7. The article adheres to appropriate reporting guidelines and community standards for data availability.

The manuscript adheres partially to appropriate reporting guidelines approved by the Ethics Committee of 85 Ramathibodi Hospital, Mahidol University. But the requirement for informed consent was waived by the Institutional Review Board
