# Supplementary material for: Correlation between clinical disease activity and interferon-γ autoantibody titers measured by inhibitory ELISA, and inflammatory biomarkers in adult-onset immunodeficiency associated with anti-interferon-γ autoantibodies
Source: PLoS One. 2026 Mar 18;21(3):e0344248. doi: 10.1371/journal.pone.0344248 (PMC12998871; doi:10.1371/journal.pone.0344248)
Supplement: Attachment — Submitted filename: Point-by-point reviews.docx [file pone.0344248.s003.docx]

This manuscript is not well prepared. It needs a major revision
1: Revise the introduction and supplement it with updated references.

**Reply:** Pages 3-5, lines 55-103 now read:

Introduction

Interferon-γ (IFN-γ) is a pivotal cytokine in the host immune response, essential for orchestrating defense mechanisms against intracellular pathogens, including non-tuberculous mycobacteria (NTM), Salmonella species, and various viral agents [1-3]. As a central component of the IFN-γ – interleukin-12 (IL-12) pathway axis, IFN-γ is primarily secreted by Th1 cells, activated cytotoxic T lymphocytes and natural killer (NK) cells. It's signaling facilitates the activation of mononuclear phagocytes, enhances antigen eradication, and modulates a pro-inflammatory cascade involving IL-12 and tumor necrosis factor-alpha (TNF-α).[4]

The criticality of the IFN-γ pathway is well-documented in both experimental and clinical settings. In experimental mouse models, a lack of IFN-γ immunity results in increased susceptibility to Mycobacterium tuberculosis, bacterial pathogens such as Listeria monocytogenes, and viral infections.[5-7] In humans, inborn errors of IFN-γ immunity-such as medelian susceptibility to mycobacterial disease (MSMD), reports variants in 21 genes that impair the production of IFN-γ. A lack of IFN-γ cytokine predisposes to mycobacterial, *Salmonella* species, and macrophage-trophic fungal infections.[8-10]

In recent years, anti-cytokine autoantibodies have emerged as a significant mechanism underlying immunodeficiency disorders [11-14]. Notably, the presence of high-affinity, neutralizing anti-interferon-γ autoantibodies (anti-IFN-γ AAbs) have been identified as the hallmark of a distinct clinical entity: adult-onset immunodeficiency associated with anti-interferon-γ autoantibodies [1, 15-17]. Patients with these autoantibodies typically present with disseminated NTM infections (approximately 80% of cases) and other opportunistic infections involving the lymph nodes, bone, lungs, skin, and bloodstream. [1, 18-23]. The clinical progression is often refractory, characterized by a fluctuation course of relapse and remission, with recurrent infections rates ranging from 18–80% [19, 20, 24, 25].

A significant challenge in the clinical management of this syndrome is the unpredictable nature of the disease.[19, 20] Currently, there is a critical lack of standardized biomarkers to accurately monitor disease activity or predict clinical flare-ups. While the measurement of anti-IFN-γ AAb titers-specifically through inhibitory ELISA-has shown promise in reflecting the neutralizing capacity of the antibodies.[25-27] Previous studies have reported an association between anti-IFN-γ AAb titers and disease activity, with an area under the receiver operating characteristic curve (AUC) of 0.8795 [28]. Anti-IFN-γ AAbs titers are not routinely available in most clinical laboratories. In clinical practice, physicians often rely on non-specific inflammatory markers, including white blood cell count (WBC), C-reactive protein (CRP), erythrocyte sedimentation rate (ESR), and hemoglobin (Hb) levels to assess infection status. [24, 25] However, the correlation between these markers and the specific pathophysiology of Anti-IFN-γ Aabs remained poorly defined. Identifying reliable, accessible biomarkers is essential for optimizing therapeutic interventions, such as the use of rituximab or other immunosuppressive therapies.[25, 26, 29] This study aimed to explore the correlation between disease activity, inhibitory ELISA titers, and the combined analysis of other inflammatory biomarkers.

2: Methods are not clear. Rewrite the methods, specify the eligibility, inclusion, exclusion, the parametes.......etc.

**Reply**: Pages 5-8, Lines 105-160 now read

**Ethical Considerations**

The study protocol was conducted as a retrospective analysis within an ongoing prospective cohort and was approved by the Ethics Committee of Ramathibodi Hospital, Mahidol University, Thailand (COA. No. MURA2025/462 and COA No. MURA2021/152). The Institutional Review Board waived the requirement for informed consent due to the retrospective nature of the analysis.

**Study Design**

This was a retrospective observational study conducted at Ramathibodi Hospital, Mahidol university, using data collected between 15 June, 2025 and 15 August, 2025.

**Study Population**

Participants were identified from the hospital-based anti-IFN-γ Aabs registry, which prospectively enrolls patients diagnosed with adult-onset immunodeficiency associated with anti-IFN-γ Aabs. The prospective cohort was established to enable longitudinal clinical and laboratory monitoring.

All patients in the cohort received standardized care within a multidisciplinary clinical framework involving infectious specialists and immunologists. Patients were routinely evaluated by both teams to develop individualized treatment plans that integrated antimicrobial therapy and immunosuppressive management when indicated. Clinical and laboratory monitoring was performed at regular intervals as part of routine care.

Laboratory evaluations included complete blood count (CBC), erythrocyte sedimention rate (ESR), C-reactive protein (CRP), liver function tests, serum creatinine (Cr), interleukin-6 (IL-6) levels and Anti-IFN-γ Aabs titers, which were typically assessed every 3-6 months. Anti-IFN-γ Aabs titers were measured in a certified laboratory using QuantiFERON-based inhibitory ELISA assay, as previously described.[31]

Eligibility Criteria

The total prospective cohort comprised 53 patients with Anti-IFN-γ Aabs-associated with immunodeficiency disease.

Inclusion criteria consisted of patients in the cohort who met the predefined criteria for either active disease or complete remission at the time of laboratory assessment.

Exclusion criteria included patients classified as being in partial remission, defined as patients who demonstrated clinical improvement following initiation of treatment but had persistent residual disease manifestations, such as unresolved lymphadenopathy. Fourteen patients were excluded based on this criterion to improve the accuracy and interpretability of the analysis. After exclusion, a total of 39 patients were included in the final analysis.

Disease Activity Classification

Disease status was classified at each follow-up visit as either active disease or remission based on clinical evaluation and physical examination performed by an infectious disease specialist and an immunologist.

Active disease was defined as the presence of new-onset fever, reactive dermatosis, or other clinical signs suggestive of ongoing or recurrent infection.

Disease remission was defined as complete clinical resolution of previously diagnosed infections with no evidence of active disease and antibiotic free.

**Data Collection**

Demographic, clinical, and laboratory data were retrieved from electronic medical records between 15 June 2025 and 15 August 2025. Laboratory data were analyzed at a single timepoint per sample. Each laboratory samples was categorized as representing either the active disease or remission phase according to the predefined disease activity criteria.

3; The results were without tables and figures???????

**Reply:**

Pages 10-11, Lines 197-201 Now read

Table 1. Demographic and clinical characteristics of patients with anti-interferon-γ autoantibodies**.**

| Characteristics | n, (%) |
| --- | --- |
| Age (years), Mean ± SD | 58.38 ± 8.55 |
| Sex |  |
| Male | 15 (38.5) |
| Female | 24 (61.5) |
| Age onset (years), Mean ± SD | 54.89 ± 9.09 |
| Active disease | 41 (59.4) |
| Thailand region of birth |  |
| Central | 18 (46.2) |
| Northeast | 13 (33.3) |
| North | 5 (12.8) |
| South | 2 (5.1) |
| West | 1 (2.6) |
| Comorbid diseases |  |
| Metabolic diseases | 24 (61.5) |
| Hypertension | 10 (26.3) |
| Dyslipidemia | 8 (20.5) |
| Diabetic mellitus | 4 (10.3) |
| Cardiovascular disease | 2 (5.1) |
| Renal disease | 7 (17.9) |
| Gastrointestinal disease | 6 (15.4) |
| Autoimmune disease | 5 (12.8) |
| Hematologic disease | 5 (12.8) |
| Neurological disease | 3 (7.7) |
| Solid malignancy | 1 (2.6) |
| Other* | 11 (28.2) |
| Clinical presentation |  |
| Lymphadenopathy | 34 (87.2) |
| Fever | 26 (66.7) |
| Reactive dermatosis | 21 (53.8) |
| Wasting | 19 (48.7) |
| Bone pain | 14 (35.9) |
| Pulmonary infection | 13 (33.3) |
| Skin infection | 7 (17.9) |
| Abdominal pain | 2 (5.1) |
| Dermatologic manifestation |  |
| Neutrophilic dermatosis | 18 (46.2) |
| Erythema nodosum | 4 (10.3) |
| Pustular | 2 (5.1) |
| Others ‡ | 1 (2.5) |
| Oppurtunistic infection † |  |
| Dissiminated nontuberculous mycobacterial infection | |
| *M.Abscessus* | 21(53.8) |
| *M.avium complex* | 8 (20.5) |
| *M.fortuitum* | 2 (5.1) |
| Other NTM species | 6 (15.4) |
| Salmonella *spp.* | 11 (28.2) |
| *Mycobacterium tubercolosis* infection | 5 (12.8) |
| Cryptococcosis | 4 (10.5) |
| Talaromycosis | 1 (2.6) |
| Others | 11 (28.2) |

† Distribution of 69 proven opportunistic pathogens diagnosed during the study period.
‡ : vasculitis

*others = thyroid disease, eye disease

Pages 12-13, lines 223-228, now read

Table 2: Laboratory biomarkers in patients with anti-interferon-γ autoantibodies: comparison between active disease and remission.

| Variables | Active | Remission | *p*-value |
| --- | --- | --- | --- |
|  | **Median (IQR]** | **Median (IQR]** |  |
| CD4+ T cell(%) | 26.75 (20.60 - 30.70) | 21.4 (19.60 - 29.20) | 0.543 |
| CD8+ T cell (%) | 30.80 (27.75 - 40.10) | 26.00 (23.10 - 36.50) | 0.234 |
| NK cell (%) | 29.10 (21.80 - 39.55) | 24.50 (15.40 - 27.60) | 0.138 |
| B cell (%) | 5.70 (2.75 - 8.60) | 10.80 (5.30 - 17.90) | 0.070 |
| Hb (g/dL) | 10.50 (9.60 - 12.20) | 12.85 (11.70 - 13.60) | 0.001* |
| WBC (cumm) | 14,830 (11,070 - 19,670) | 7,380 (5,910 - 8,710) | <0.001* |
| ESR | 65.00 (44.00 - 79.50) | 30.50 (12.50- 50.00) | <0.001* |
| CRP | 61.66 (22.29 - 96.15) | 3.98 (1.16 - 12.08) | <0.001* |
| IL-6 | 17.60 (8.40 – 38.00) | 4.50 (2.35 - 10.90) | 0.001* |
| ALP | 150 (91 - 270) | 88 (83 - 120) | 0.005* |
| Anti-interferon-γ titer | 1:100,000  (1:10,000 – 1:100,000) | 1:5,000  (1:5,000 – 1:10,000) | <0.001* |

**P* < 0.05

ALP; alkaline phosphatase, Anti-IFN γ Aabs titers; Anti-interferon-gamma autoantibodies titers, CRP; C-reactive protein, ESR; erythrocyte sedimentary rate, Hb; hemoglobin, NK cell; natural killer cell, WBC; White blood cell count, IL-6; interleukin 6 level

Pages 13-14, lines 234-239, now read

**Table 3.** Diagnostic performance metrics of Anti-IFN-γ autoantibody titers and inflammatory markers.

| Parameter | AUC  (95% CI) | Sensitivity, %  (95% CI) | Specificity, %  (95% CI) | LR+  (95% CI) | LR−  (95% CI) | PPV %  (95% CI) | NPV %  (95% CI) | Accuracy %  (95% CI) |
| --- | --- | --- | --- | --- | --- | --- | --- | --- |
| Anti–IFN-γ AAbs ≥ 50,000 | **0.893**  (0.821–0.965) | **65.9**  (49.4–79.9) | **92.9**  (76.5–99.1) | **9.22**  (2.38–35.7) | **0.37**  (0.24–0.57) | **93.1**  (77.2–99.2) | **65.0**  (48.3–79.4) | **76.8**  (65.1–86.1) |
| Anti–IFN-γ AAbs ≥ 10,000 | **0.893**  (0.821–0.965) | **97.6**  (87.1–99.9) | **53.6**  (33.9–72.5) | **2.10**  (1.41–3.14) | **0.05**  (0.01–0.33) | **75.5**  (61.7–86.2) | **93.8**  (69.8–99.8) | **79.7**  (68.3–88.4) |
| WBC  (≥10,455 cells/µL) | **0.926**  (0.865-0.987) | **82.9**  (67.9-92.8) | **92.9**  (76.5-99.1) | **11.6**  3.03-44.5 | **0.184**  (0.0929-0.364) | **94.4**  (81.3-99.3) | **78.8**  (61.1-91.0) | **87**  (77.0-94.0) |
| Hemoglobin  (≤11.45 g/dL) | **0.736**  (0.614–0.859) | **70.7**  (54.5–83.9) | **82.1**  (63.1–93.9) | **3.96**  (1.75–8.98) | **0.36**  (0.22–0.59) | **85.3**  (68.9–95.0) | **65.7**  (47.8–80.9) | **75.4**  (63.5–84.9) |
| ESR  (≥45 mm/hr) | **0.750**  (0.628–0.872) | **75.0**  (58.8–87.3) | **75.0**  (55.1–89.3) | **3.00**  (1.54–5.84) | **0.33**  (0.19–0.59) | **81.1**  (64.8–92.0) | **67.7**  (48.6–83.3) | **75.0**  (63.0–84.7) |
| CRP  (≥18 mg/L) | **0.864**  (0.774–0.954) | **80.0**  (64.4–90.9) | **88.9**  (70.8–97.6) | **7.20**  (2.45–21.2) | **0.23**  (0.12–0.42) | **91.4**  (76.9–98.2) | **75.0**  (56.6–88.5) | **83.6**  (72.5–91.5) |
| IL-6  ((≥16 pg/mL) | **0.781**  (0.652–0.910) | **59.3**  (38.8–77.6) | **91.7**  (73.0–99.0) | **7.11**  (1.82–27.8) | **0.44**  (0.28–0.71) | **88.9**  (65.3–98.6) | **66.7**  (48.2–82.0) | **74.5** (60.4–85.7) |

Anti-IFN γ Aabs titers; Anti-interferon-gamma autoantibodies titers, AUC; area under the curve, CRP; C-reactive protein, ESR; erythrocyte sedimentary rate, Hb; hemoglobin, NPV; negative predictive value, PPV; positive predictive value, LR+; positive likelihood ratio, LR-; negative likelihood ratio, WBC; White blood cell count, IL-6; interleukin 6 level

Pages 13-14, lines 241-243, now read

Figure 1. ROC analysis of laboratory biomarker cut-off values for predicting active disease.

Pages 17, lines 292-296, now read

Table 4. Performance of combined laboratory parameters in predicting disease.

| Biomarker combination | AUC  (95% CI) | Sensitivity %  (95% CI) | Specificity %  (95% CI) | LR+  (95% CI) | LR−  (95% CI) | PPV %  (95% CI) | NPV %  (95% CI) | Accuracy %  (95% CI) |
| --- | --- | --- | --- | --- | --- | --- | --- | --- |
| Anti–IFN-γ AAbs + ESR + CRP + WBC + IL-6 | **0.903**  (0.808–0.997) | **88.9**  (70.8–97.6) | **91.7**  (73.0–99.0) | **10.7**  (2.81–40.5) | **0.12**  (0.04–0.36) | **92.3**  (74.9–99.1) | **88.0**  (68.8–97.5) | **90.2**  (78.6–96.7) |
| Anti–IFN-γ AAbs + ESR + CRP | **0.869**  (0.775–0.964) | **85.0**  (70.2–94.3) | **88.9**  (70.8–97.6) | **7.65**  (2.61–22.4) | **0.17**  (0.08–0.36) | **91.9**  (78.1–98.3) | **80.0**  (61.4–92.3) | **86.6**  (76.0–93.7) |
| Anti–IFN-γ AAbs + ESR + CRP + WBC | **0.913**  (0.834–0.992) | **90.0**  (76.3–97.2) | **92.6**  (75.7–99.1) | **12.2**  (3.19–46.3) | **0.11**  (0.04–0.28) | **94.7**  (82.3–99.4) | **86.2**  (68.3–96.1) | **91.0**  (81.5–96.6) |
| CRP + ESR + WBC | **0.869**  (0.775–0.964) | **85.0**  (70.2–94.3) | **88.9**  (70.8–97.6) | **7.65**  (2.61–22.4) | **0.17**  (0.08–0.36) | **91.9**  (78.1–98.3) | **80.0**  (61.4–92.3) | **86.6**  (76.0–93.7) |
| CRP + ESR + IL-6 | **0.789**  (0.660–0.919) | **70.4**  (49.8–86.2) | **87.5**  (67.6–97.3) | **5.63**  (1.90–16.7) | **0.34**  (0.19–0.62) | **86.4**  (65.1–97.1) | **72.4**  (52.8–87.3) | **78.4**  (64.7–88.7) |

Anti-IFN γ Aabs titers; Anti-interferon-gamma autoantibodies titers, AUC; area under the curve, CRP; C-reactive protein, ESR; erythrocyte sedimentary rate, Hb; hemoglobin, IL-6; interleukin 6 level, LR+; positive likelihood ratio, LR-; negative likelihood ratio, NPV; negative predictive value, PPV; positive predictive value, WBC; White blood cell count

4: Rewrite the discussion. The authors added only one reference to the discussion????

**Reply:** Pages 19-23, lines 340-424, Reference n=13, now read

The role of anti-IFN-γ AAbs in immunodeficiency and infectious disease, is well established.[19, 32] Patients with anti-IFN-γ AAbs frequently experience recurrent and relapsing infection, particularly due to NTM infection and other intracellular organisms. [1, 15, 16, 18] Treatment regimens and duration are determined by the causative pathogens and the extent of organs involvement.[33] Therefore, close monitoring of clinical response and laboratory biomarkers is essential to optimize treatment outcome.

Previous studies reported that serum anti-IFN-γ Aabs titer are a potential biomarker for evaluating disease activity in AIGA patients. anti-IFN-γ Aabs titers fluctuate over the course of the disease, the antibody level decrease when the disease improves with treatment and increase during active disease.[28, 29, 34] Nithichanon et al. explored the efficacy of anti-IFN-γ AAb titers in distinguishing between patients with active and inactive NTM infection, and demonstrated a significant association between anti-IFN-γ AAb titers ,with a cut-off inhibition titer ≥ 1:5,000 and active disease, AUC=0.8795.[29]

In our study, anti-IFN-γ AAb titers exhibited strong diagnostic performance in differentiating active disease from remission as definition, with an AUC of 0.893. At a cut-off of >1:50,000, the titers demonstrated high specificity (92.90%) and a moderate sensitivity (65.90%), supporting their utility in confirming disease activity. Lowering the cut-off to ≥1:10,000 increased sensitivity but reduced specificity, highlighting the trade-off between sensitivity and specificity. Measurement of anti-IFN-γ AAb titers by inhibitory ELISA may further enhance the diagnostic workflow by directly assessing functional neutralization of IFN-γ. This assay may complement indirect ELISA, particularly in cases with borderline titers or discordant clinical features. Although anti-IFN-γ Aabs titers are a high-performance biomarker reflecting disease status, their use may be limited in some centers due to resource constrains.

Other laboratory biomarkers have been investigated for disease monitoring in Anti-IFN-γ Aabs patients. A prospective cohort study comparing AIGA-positive and AIGA-negative patients with mycobacterium tuberculosis infection reported a significantly highly levels of WBC, neutrophils, monocytes, CRP, ESR, globulin and immunoglobulin G level in AIGA-positive (p<0.05).[35] Laisuan et al.[26] reported changes in biomarkers among AIGA patients receiving adjunctive immunosuppressive therapy, with remission associated with decreased levels of WBC, ESR and CRP compared with active disease.[26] Similar trends were observed by Angkasekeinai et al.[25], who demonstrated that CRP, ESR and WBC were meaningful biomarkers for categorizing disease activity in patients with anti-IFN-γ Aabs patients.

In this study, additional inflammatory biomarkers including Hb, WBC, ESR, CRP, and IL-6 were evaluated for diagnostic performance. Among individual markers, WBC count exhibited the strongest discriminatory power, with an AUC of 0.926 and a high LR+ of 11.61. WBC counts were significantly elevated during periods of active disease, supporting their utility in monitoring disease activity in patients with anti-IFN-γ AAbs.

CRP and ESR are well-established acute-phase reactants commonly used to monitor inflammation and demonstrated moderate to good diagnostic utility in this study. CRP had an AUC of 0.864, with an optimal cut-off of ≥18 mg/L, achieving good sensitivity (80.00%) and specificity (88.90%). ESR exhibited moderate utility, with an AUC of 0.750. This findings are consistent with the known roles of ESR and CRP levels in reflecting inflammatory responses to infection in patients with anti-IFN-γ Aabs.[26] IL-6, another inflammatory cytokine, also correlated with disease activity demonstrated good specificity with cut off ≥ 16 pg/mL, although to a lesser extent than CRP and ESR, with an AUC of 0.789. These results suggest that IL-6 may serve as a supplementary biomarker but is less reliable than other inflammatory biomarkers in use alone. However, its availability may be limited in routine clinical practice.

Comorbid conditions in AIGA patients, including concurrent infections, anemia, hepatic diseases, renal impairment, and other inflammatory states, may influence biomarker level independently of disease activity. These factors may result in either overestimation or underestimation of biomarker performance when distinguishing active disease from remission. Nevertheless, the combined use of multiple biomarkers may improve overall diagnostic accuracy in determining disease activity by mitigating the limitations of individual markers.

The combination of biomarkers improved diagnostic performance. The addition of anti-IFN-γ AAb titers and WBC increased both the sensitivity and specificity of the diagnostic models. The combination of anti-IFN-γ AAb titers, ESR, CRP, and WBC count demonstrated excellent performance (AUC = 0.913), with high sensitivity (90.00%), specificity (92.60%), and PPV (94.70%), providing the highest overall diagnostic accuracy among the tested panels. This underscores the benefit of integrating multiple laboratory biomarkers for optimal disease monitoring. Although, the combination of CRP, ESR, and WBC was less accurate (AUC = 0.869), it still exhibited moderate diagnostic performance, suggesting that this simpler panel may serve as a practical and more accessible option for routine clinical monitoring, particularly in resource-limited settings.

Despite the promising results, several limitations should be acknowledged. The retrospective design and relatively small sample size may have limited the statistical power of specific analyses. Additionally, although the biomarkers evaluated provide valuable diagnostic information, they do not fully capture the complex immunological mechanisms underlying anti-IFN-γ AAb-associated immunodeficiency. Prospective studies with larger, more diverse cohorts are needed to validate the clinical utility of these biomarkers. Future research should also examine longitudinal changes in biomarker levels to assess their predictive value for disease recurrence, progression, or flare-ups.

Reviewer #1: The analysis isn´t enough descriptive. Previous comorbidity pathologic conditions doesn´t mention if earliest disease manifestations can be related to the so called initial adult-onset immunodeficiency nor if there are previous treatments achieved during early comorbidities, evolution or manifestations of disease. Analysis doesn´t mention changes for expresión of activation markers among immunocompetent population nor if there is a shift from a Th1/Th2, inflammatory/anti-inflammatory profile of population, and even in the secretion of cytokine profiles of subpopulations. It would be appropriate to know if such immunologic adult-onset disease parameters are related to clinical course changes from active to remission of disease. There is also a lack of other inflammatory and/or fibrin- or coagulation-related factors, such as ferritin, D-dimer, PLT and thrombin-related times which would perpetuate inflammatory profiles. In my opinión, there are some issues that remain to be defined in a context-dependent manner as in maintaining active treatment in order to avoid progression after ending treatment. In summary changes in remission states have their own positive clinical characteristics at remission contrary to those at initial adult-onset diagnosis but it means to check clinical course of disease along time in order to maintain anti-inflammatory changes.

**Reply:**

We sincerely thank the reviewer for the thoughtful and insightful comments. We agree that a more detailed immunological characterization, including comorbid conditions, early disease manifestations, activation markers, T-helper cell profiles, cytokine subpopulations, and coagulation-related parameters (e.g., ferritin, D-dimer, platelet count, and thrombin-related indices), would provide valuable mechanistic insights into disease evolution and remission in adult-onset immunodeficiency associated with anti-IFN-γ autoantibodies.

However, we would like to clarify that the primary objective of the present study was to evaluate clinically applicable biomarkers for monitoring disease activity in real-world clinical practice. This study was designed as a retrospective clinical analysis nested within an ongoing prospective cohort, and therefore focused on routinely available laboratory parameters and clinical disease status rather than in-depth immunophenotyping or mechanistic immune profiling.

Importantly, this work builds upon our previous prospective studies, in which we demonstrated dynamic changes in conventional inflammatory biomarkers (including WBC count, ESR, CRP, and IL-6) between active and remission states, particularly in patients receiving adjunctive immunosuppressive therapy. Those earlier studies provided foundational evidence supporting the clinical relevance of these markers, and the current study aimed to further validate and integrate these findings into practical diagnostic panels.

We fully acknowledge that the absence of detailed immune activation markers, Th1/Th2 profiling, and broader cytokine or coagulation assessments represents a limitation of the present study. These advanced immunological investigations were beyond the scope of the current clinical dataset and were not routinely available in our real-world cohort. Nevertheless, we believe that the strength of this study lies in its translational focus, emphasizing accessible and reproducible biomarkers that can be readily applied in routine clinical settings, particularly in resource-limited environments.

Future prospective studies incorporating comprehensive immunological and molecular profiling will be essential to elucidate the underlying mechanisms of immune dysregulation and to better understand how immunological shifts correlate with long-term clinical outcomes and remission maintenance.

Reviewer #2: In this manuscript, the authors investigate the correlation between anti–interferon‑γ autoantibody titers measured by inhibitory ELISA and clinical disease activity, as well as inflammatory biomarkers in adult‑onset immunodeficiency. The topic is clinically important, especially given the high prevalence of anti‑IFN‑γ autoantibody–associated immunodeficiency in Southeast Asia and the ongoing need for reliable biomarkers for disease monitoring. The study is generally well-written and contains meaningful data; however, several major and minor issues should be addressed before the manuscript can be considered for publication.

**Reply**: We sincerely thank the reviewer for the positive and constructive comments regarding the clinical relevance of our study and for recognizing the importance of this topic, particularly in the Southeast Asian context. We greatly appreciate the reviewer’s careful evaluation of the manuscript and the valuable suggestions provided.

We acknowledge that several major and minor issues require clarification and improvement. We have carefully addressed each point raised and have revised the manuscript accordingly to strengthen the methodological transparency, data presentation, and interpretation of the findings. Specifically, we have expanded the Methods section, clarified inclusion and exclusion criteria, added missing information on clinical characteristics, and provided additional statistical details where appropriate.

We believe that these revisions have substantially improved the scientific rigor and clarity of the manuscript and hope that the revised version now adequately addresses the reviewer’s concerns. We are grateful for the reviewer’s insightful feedback, which has significantly contributed to enhancing the overall quality of our work.

**Major Comments**
1. The manuscript repeatedly refers to Table 1-4 and Figure 1, but these materials do not appear in the provided document. This significantly impairs the ability to evaluate data presentation, statistical validity, interpretation of ROC curves and diagnostic performance. Therefore, please ensure that all tables and figures referenced in the text are included, properly formatted, and placed within the main manuscript, or uploaded as separate supporting files.

Reply: We sincerely apologize for this oversight and thank the reviewer for pointing out the missing tables and figure. We have now carefully reviewed the submission and ensured that all referenced Tables 1-4 and Figure 1 are included in the revised manuscript.

Pages 10-11, Lines 197-201 Now read

Table 1. Demographic and clinical characteristics of patients with anti-interferon-γ autoantibodies**.**

| Characteristics | n, (%) |
| --- | --- |
| Age (years), Mean ± SD | 58.38 ± 8.55 |
| Sex |  |
| Male | 15 (38.5) |
| Female | 24 (61.5) |
| Age onset (years), Mean ± SD | 54.89 ± 9.09 |
| Active disease | 41 (59.4) |
| Thailand region of birth |  |
| Central | 18 (46.2) |
| Northeast | 13 (33.3) |
| North | 5 (12.8) |
| South | 2 (5.1) |
| West | 1 (2.6) |
| Comorbid diseases |  |
| Metabolic diseases | 24 (61.5) |
| Hypertension | 10 (26.3) |
| Dyslipidemia | 8 (20.5) |
| Diabetic mellitus | 4 (10.3) |
| Cardiovascular disease | 2 (5.1) |
| Renal disease | 7 (17.9) |
| Gastrointestinal disease | 6 (15.4) |
| Autoimmune disease | 5 (12.8) |
| Hematologic disease | 5 (12.8) |
| Neurological disease | 3 (7.7) |
| Solid malignancy | 1 (2.6) |
| Other* | 11 (28.2) |
| Clinical presentation |  |
| Lymphadenopathy | 34 (87.2) |
| Fever | 26 (66.7) |
| Reactive dermatosis | 21 (53.8) |
| Wasting | 19 (48.7) |
| Bone pain | 14 (35.9) |
| Pulmonary infection | 13 (33.3) |
| Skin infection | 7 (17.9) |
| Abdominal pain | 2 (5.1) |
| Dermatologic manifestation |  |
| Neutrophilic dermatosis | 18 (46.2) |
| Erythema nodosum | 4 (10.3) |
| Pustular | 2 (5.1) |
| Others ‡ | 1 (2.5) |
| Oppurtunistic infection † |  |
| Dissiminated nontuberculous mycobacterial infection | |
| *M.Abscessus* | 21(53.8) |
| *M.avium complex* | 8 (20.5) |
| *M.fortuitum* | 2 (5.1) |
| Other NTM species | 6 (15.4) |
| Salmonella *spp.* | 11 (28.2) |
| *Mycobacterium tubercolosis* infection | 5 (12.8) |
| Cryptococcosis | 4 (10.5) |
| Talaromycosis | 1 (2.6) |
| Others | 11 (28.2) |

† Distribution of 69 proven opportunistic pathogens diagnosed during the study period.
‡ : vasculitis

*others = thyroid disease, eye disease

Pages 12-13, lines 223-228, now read

Table 2: Laboratory biomarkers in patients with anti-interferon-γ autoantibodies: comparison between active disease and remission.

| Variables | Active | Remission | *p*-value |
| --- | --- | --- | --- |
|  | **Median (IQR]** | **Median (IQR]** |  |
| CD4+ T cell(%) | 26.75 (20.60 - 30.70) | 21.4 (19.60 - 29.20) | 0.543 |
| CD8+ T cell (%) | 30.80 (27.75 - 40.10) | 26.00 (23.10 - 36.50) | 0.234 |
| NK cell (%) | 29.10 (21.80 - 39.55) | 24.50 (15.40 - 27.60) | 0.138 |
| B cell (%) | 5.70 (2.75 - 8.60) | 10.80 (5.30 - 17.90) | 0.070 |
| Hb (g/dL) | 10.50 (9.60 - 12.20) | 12.85 (11.70 - 13.60) | 0.001* |
| WBC (cumm) | 14,830 (11,070 - 19,670) | 7,380 (5,910 - 8,710) | <0.001* |
| ESR | 65.00 (44.00 - 79.50) | 30.50 (12.50- 50.00) | <0.001* |
| CRP | 61.66 (22.29 - 96.15) | 3.98 (1.16 - 12.08) | <0.001* |
| IL-6 | 17.60 (8.40 – 38.00) | 4.50 (2.35 - 10.90) | 0.001* |
| ALP | 150 (91 - 270) | 88 (83 - 120) | 0.005* |
| Anti-interferon-γ titer | 1:100,000  (1:10,000 – 1:100,000) | 1:5,000  (1:5,000 – 1:10,000) | <0.001* |

**P* < 0.05

ALP; alkaline phosphatase, Anti-IFN γ Aabs titers; Anti-interferon-gamma autoantibodies titers, CRP; C-reactive protein, ESR; erythrocyte sedimentary rate, Hb; hemoglobin, NK cell; natural killer cell, WBC; White blood cell count, IL-6; interleukin 6 level

Pages 13-14, lines 234-239, now read

**Table 3.** Diagnostic performance metrics of Anti-IFN-γ autoantibody titers and inflammatory markers.

| Parameter | AUC  (95% CI) | Sensitivity, %  (95% CI) | Specificity, %  (95% CI) | LR+  (95% CI) | LR−  (95% CI) | PPV %  (95% CI) | NPV %  (95% CI) | Accuracy %  (95% CI) |
| --- | --- | --- | --- | --- | --- | --- | --- | --- |
| Anti–IFN-γ AAbs ≥ 50,000 | **0.893**  (0.821–0.965) | **65.9**  (49.4–79.9) | **92.9**  (76.5–99.1) | **9.22**  (2.38–35.7) | **0.37**  (0.24–0.57) | **93.1**  (77.2–99.2) | **65.0**  (48.3–79.4) | **76.8**  (65.1–86.1) |
| Anti–IFN-γ AAbs ≥ 10,000 | **0.893**  (0.821–0.965) | **97.6**  (87.1–99.9) | **53.6**  (33.9–72.5) | **2.10**  (1.41–3.14) | **0.05**  (0.01–0.33) | **75.5**  (61.7–86.2) | **93.8**  (69.8–99.8) | **79.7**  (68.3–88.4) |
| WBC | **0.926**  (0.865-0.987) | **82.9**  (67.9-92.8) | **92.9**  (76.5-99.1) | **11.6**  3.03-44.5 | **0.184**  (0.0929-0.364) | **94.4**  (81.3-99.3) | **78.8**  (61.1-91.0) | **87**  (77.0-94.0) |
| Hemoglobin | **0.736**  (0.614–0.859) | **70.7**  (54.5–83.9) | **82.1**  (63.1–93.9) | **3.96**  (1.75–8.98) | **0.36**  (0.22–0.59) | **85.3**  (68.9–95.0) | **65.7**  (47.8–80.9) | **75.4**  (63.5–84.9) |
| ESR | **0.750**  (0.628–0.872) | **75.0**  (58.8–87.3) | **75.0**  (55.1–89.3) | **3.00**  (1.54–5.84) | **0.33**  (0.19–0.59) | **81.1**  (64.8–92.0) | **67.7**  (48.6–83.3) | **75.0**  (63.0–84.7) |
| CRP | **0.864**  (0.774–0.954) | **80.0**  (64.4–90.9) | **88.9**  (70.8–97.6) | **7.20**  (2.45–21.2) | **0.23**  (0.12–0.42) | **91.4**  (76.9–98.2) | **75.0**  (56.6–88.5) | **83.6**  (72.5–91.5) |
| IL-6 | **0.781**  (0.652–0.910) | **59.3**  (38.8–77.6) | **91.7**  (73.0–99.0) | **7.11**  (1.82–27.8) | **0.44**  (0.28–0.71) | **88.9**  (65.3–98.6) | **66.7**  (48.2–82.0) | **74.5** (60.4–85.7) |

Anti-IFN γ Aabs titers; Anti-interferon-gamma autoantibodies titers, AUC; area under the curve, CRP; C-reactive protein, ESR; erythrocyte sedimentary rate, Hb; hemoglobin, NPV; negative predictive value, PPV; positive predictive value, LR+; positive likelihood ratio, LR-; negative likelihood ratio, WBC; White blood cell count, IL-6; interleukin 6 level

Pages 14, lines 241-243, now read

Figure 1. ROC analysis of laboratory biomarker cut-off values for predicting active disease.

Pages 17, lines 292-296, now read

Table 4. Performance of combined laboratory parameters in predicting disease.

| Biomarker combination | AUC  (95% CI) | Sensitivity %  (95% CI) | Specificity %  (95% CI) | LR+  (95% CI) | LR−  (95% CI) | PPV %  (95% CI) | NPV %  (95% CI) | Accuracy %  (95% CI) |
| --- | --- | --- | --- | --- | --- | --- | --- | --- |
| Anti–IFN-γ AAbs + ESR + CRP + WBC + IL-6 | **0.903**  (0.808–0.997) | **88.9**  (70.8–97.6) | **91.7**  (73.0–99.0) | **10.7**  (2.81–40.5) | **0.12**  (0.04–0.36) | **92.3**  (74.9–99.1) | **88.0**  (68.8–97.5) | **90.2**  (78.6–96.7) |
| Anti–IFN-γ AAbs + ESR + CRP | **0.869**  (0.775–0.964) | **85.0**  (70.2–94.3) | **88.9**  (70.8–97.6) | **7.65**  (2.61–22.4) | **0.17**  (0.08–0.36) | **91.9**  (78.1–98.3) | **80.0**  (61.4–92.3) | **86.6**  (76.0–93.7) |
| Anti–IFN-γ AAbs + ESR + CRP + WBC | **0.913**  (0.834–0.992) | **90.0**  (76.3–97.2) | **92.6**  (75.7–99.1) | **12.2**  (3.19–46.3) | **0.11**  (0.04–0.28) | **94.7**  (82.3–99.4) | **86.2**  (68.3–96.1) | **91.0**  (81.5–96.6) |
| CRP + ESR + WBC | **0.869**  (0.775–0.964) | **85.0**  (70.2–94.3) | **88.9**  (70.8–97.6) | **7.65**  (2.61–22.4) | **0.17**  (0.08–0.36) | **91.9**  (78.1–98.3) | **80.0**  (61.4–92.3) | **86.6**  (76.0–93.7) |
| CRP + ESR + IL-6 | **0.789**  (0.660–0.919) | **70.4**  (49.8–86.2) | **87.5**  (67.6–97.3) | **5.63**  (1.90–16.7) | **0.34**  (0.19–0.62) | **86.4**  (65.1–97.1) | **72.4**  (52.8–87.3) | **78.4**  (64.7–88.7) |

Anti-IFN γ Aabs titers; Anti-interferon-gamma autoantibodies titers, AUC; area under the curve, CRP; C-reactive protein, ESR; erythrocyte sedimentary rate, Hb; hemoglobin, IL-6; interleukin 6 level, LR+; positive likelihood ratio, LR-; negative likelihood ratio, NPV; negative predictive value, PPV; positive predictive value, WBC; White blood cell count

2. The study is described as retrospective, nested within a prospective cohort, but essential methodological details require clarification. Were samples collected and analyzed at uniform time points for all patients? Were the biomarkers measured at the same visit as disease activity assessment? These clarifications are needed to ensure internal validity and avoid bias.

Reply: Pages 5-9, Lines 105-160 now read

“**Ethical Considerations**

The study protocol was conducted as a retrospective analysis within an ongoing prospective cohort and was approved by the Ethics Committee of Ramathibodi Hospital, Mahidol University, Thailand (COA. No. MURA2025/462 and COA No. MURA2021/152). The Institutional Review Board waived the requirement for informed consent due to the retrospective nature of the analysis.

**Study Design**

This was a retrospective observational study conducted at Ramathibodi Hospital, Mahidol university, using data collected between 15 June, 2025 and 15 August, 2025.

**Study Population**

Participants were identified from the hospital-based anti-IFN-γ Aabs registry, which prospectively enrolls patients diagnosed with adult-onset immunodeficiency associated with anti-IFN-γ Aabs. The prospective cohort was established to enable longitudinal clinical and laboratory monitoring.

All patients in the cohort received standardized care within a multidisciplinary clinical framework involving infectious specialists and immunologists. Patients were routinely evaluated by both teams to develop individualized treatment plans that integrated antimicrobial therapy and immunosuppressive management when indicated. Clinical and laboratory monitoring was performed at regular intervals as part of routine care.

Laboratory evaluations included complete blood count (CBC), erythrocyte sedimention rate (ESR) , C-reactive protein (CRP), liver function tests, serum creatinine (Cr), interleukin-6 (IL-6) levels and Anti-IFN-γ Aabs titers, which were typically assessed every 3-6 months.

**Eligibility Criteria**

The total prospective cohort comprised 53 patients with Anti-IFN-γ Aabs-associated with immunodeficiency disease.

Inclusion criteria consisted of patients in the cohort who met the predefined criteria for either active disease or complete remission at the time of laboratory assessment.

Exclusion criteria included patients classified as being in partial remission, defined as patients who demonstrated clinical improvement following initiation of treatment but had persistent residual disease manifestations, such as unresolved lymphadenopathy. Fourteen patients were excluded based on this criterion to improve the accuracy and interpretability of the analysis. After exclusion, a total of 39 patients were included in the final analysis.

Disease Activity Classification

Disease status was classified at each follow-up visit as either active disease or remission based on clinical evaluation and physical examination performed by an infectious disease specialist and an immunologist.

Active disease was defined as the presence of new-onset fever, reactive dermatosis, or other clinical signs suggestive of ongoing or recurrent infection.

Disease remission was defined as complete clinical resolution of previously diagnosed infections with no evidence of active disease.

**Data Collection**

Demographic, clinical, and laboratory data were retrieved from electronic medical records between 15 June 2025 and 15 August 2025. Laboratory data were analyzed at a single timepoint per sample. Each laboratory samples was categorized as representing either the active disease or remission phase according to the predefined disease activity criteria.”

3. Inclusion and exclusion criteria need further explanation. Whether all consecutive patients with anti‑IFN‑γ auto Abs were included. Whether disease activity distribution (40 active vs 29 remission samples) might reflect selection bias. Clarification is required to validate the generalizability of the results.

**Reply:** Pages 7-8, lines 133-154, now read

Eligibility Criteria

The total prospective cohort comprised 53 patients with Anti-IFN-γ Aabs-associated with immunodeficiency disease.

Inclusion criteria consisted of patients in the cohort who met the predefined criteria for either active disease or complete remission at the time of laboratory assessment.

Exclusion criteria included patients classified as being in partial remission, defined as patients who demonstrated clinical improvement following initiation of treatment but had persistent residual disease manifestations, such as unresolved lymphadenopathy. Fourteen patients were excluded based on this criterion to improve the accuracy and interpretability of the analysis. After exclusion, a total of 39 patients were included in the final analysis.

Disease Activity Classification

Disease status was classified at each follow-up visit as either active disease or remission based on clinical evaluation and physical examination performed by an infectious disease specialist and an immunologist.

Active disease was defined as the presence of new-onset fever, reactive dermatosis, or other clinical signs suggestive of ongoing or recurrent infection.

Disease remission was defined as complete clinical resolution of previously diagnosed infections with no evidence of active disease and antibiotic free.

4. There is no information on duration of illness, prior antimicrobial or immunosuppressive therapy, timing of relapse/remission cycles. These variables could significantly influence biomarkers (e.g., CRP, ESR, IL‑6) and may confound interpretation of disease activity. Therefore, this information should be included.

**Reply:** Thank you for this insightful comment. We agree that disease duration, prior antimicrobial or immunosuppressive therapy, and relapse-remission timing may influence inflammatory biomarkers. However, the aim of this study was to assess the cross-sectional association between biomarkers and clinically defined disease activity at the time of sampling. Owing to the retrospective design and heterogeneity of treatments and follow-up, these variables could not be uniformly analyzed. This limitation has been acknowledged, and future prospective longitudinal studies are needed to address these factors in greater detail. Opportunistic infections, clinical presentations, and comorbidities of the study population are summarized in Table 1.

5. The Methods section requires further clarification and expansion, particularly regarding Inhibitory ELISA methodology, cytokine assays and immune cell population analysis. At present, essential methodological details are missing, which limits the reproducibility and interpretability of the results.

**Reply:** Pages 6-7, lines 127-132 now read

Laboratory evaluations included complete blood count (CBC), erythrocyte sedimention rate (ESR), C-reactive protein (CRP), liver function tests, serum creatinine (Cr), interleukin-6 (IL-6) levels and Anti-IFN-γ Aabs titers, which were typically assessed every 3-6 months. Anti-IFN-γ Aabs titers were measured in a certified laboratory using QuantiFERON-based inhibitory ELISA assay, as previously described.[31]

6. The manuscript reports AUC, cutoffs, sensitivity, specificity, etc., but confidence intervals (CIs) are missing for many key metrics. CIs are essential for interpreting robustness and reproducibility. Logistic regression analysis is briefly mentioned, but the regression model structure, variable selection strategy, model diagnostics (multicollinearity, goodness‑of‑fit), are insufficiently described.

**Reply:** Table 3 now read

| Parameter | AUC  (95% CI) | Sensitivity, %  (95% CI) | Specificity, %  (95% CI) | LR+  (95% CI) | LR−  (95% CI) | PPV %  (95% CI) | NPV %  (95% CI) | Accuracy %  (95% CI) |
| --- | --- | --- | --- | --- | --- | --- | --- | --- |
| Anti–IFN-γ AAbs ≥ 50,000 | **0.893**  (0.821–0.965) | **65.9**  (49.4–79.9) | **92.9**  (76.5–99.1) | **9.22**  (2.38–35.7) | **0.37**  (0.24–0.57) | **93.1**  (77.2–99.2) | **65.0**  (48.3–79.4) | **76.8**  (65.1–86.1) |
| Anti–IFN-γ AAbs ≥ 10,000 | **0.893**  (0.821–0.965) | **97.6**  (87.1–99.9) | **53.6**  (33.9–72.5) | **2.10**  (1.41–3.14) | **0.05**  (0.01–0.33) | **75.5**  (61.7–86.2) | **93.8**  (69.8–99.8) | **79.7**  (68.3–88.4) |
| WBC | **0.926**  (0.865-0.987) | **82.9**  (67.9-92.8) | **92.9**  (76.5-99.1) | **11.6**  3.03-44.5 | **0.184**  (0.0929-0.364) | **94.4**  (81.3-99.3) | **78.8**  (61.1-91.0) | **87**  (77.0-94.0) |
| Hemoglobin | **0.736**  (0.614–0.859) | **70.7**  (54.5–83.9) | **82.1**  (63.1–93.9) | **3.96**  (1.75–8.98) | **0.36**  (0.22–0.59) | **85.3**  (68.9–95.0) | **65.7**  (47.8–80.9) | **75.4**  (63.5–84.9) |
| ESR | **0.750**  (0.628–0.872) | **75.0**  (58.8–87.3) | **75.0**  (55.1–89.3) | **3.00**  (1.54–5.84) | **0.33**  (0.19–0.59) | **81.1**  (64.8–92.0) | **67.7**  (48.6–83.3) | **75.0**  (63.0–84.7) |
| CRP | **0.864**  (0.774–0.954) | **80.0**  (64.4–90.9) | **88.9**  (70.8–97.6) | **7.20**  (2.45–21.2) | **0.23**  (0.12–0.42) | **91.4**  (76.9–98.2) | **75.0**  (56.6–88.5) | **83.6**  (72.5–91.5) |
| IL-6 | **0.781**  (0.652–0.910) | **59.3**  (38.8–77.6) | **91.7**  (73.0–99.0) | **7.11**  (1.82–27.8) | **0.44**  (0.28–0.71) | **88.9**  (65.3–98.6) | **66.7**  (48.2–82.0) | **74.5** (60.4–85.7) |

Table 4 now read

| Biomarker combination | AUC  (95% CI) | Sensitivity %  (95% CI) | Specificity %  (95% CI) | LR+  (95% CI) | LR−  (95% CI) | PPV %  (95% CI) | NPV %  (95% CI) | Accuracy %  (95% CI) |
| --- | --- | --- | --- | --- | --- | --- | --- | --- |
| Anti–IFN-γ AAbs + ESR + CRP + WBC + IL-6 | **0.903**  (0.808–0.997) | **88.9**  (70.8–97.6) | **91.7**  (73.0–99.0) | **10.7**  (2.81–40.5) | **0.12**  (0.04–0.36) | **92.3**  (74.9–99.1) | **88.0**  (68.8–97.5) | **90.2**  (78.6–96.7) |
| Anti–IFN-γ AAbs + ESR + CRP | **0.869**  (0.775–0.964) | **85.0**  (70.2–94.3) | **88.9**  (70.8–97.6) | **7.65**  (2.61–22.4) | **0.17**  (0.08–0.36) | **91.9**  (78.1–98.3) | **80.0**  (61.4–92.3) | **86.6**  (76.0–93.7) |
| Anti–IFN-γ AAbs + ESR + CRP + WBC | **0.913**  (0.834–0.992) | **90.0**  (76.3–97.2) | **92.6**  (75.7–99.1) | **12.2**  (3.19–46.3) | **0.11**  (0.04–0.28) | **94.7**  (82.3–99.4) | **86.2**  (68.3–96.1) | **91.0**  (81.5–96.6) |
| CRP + ESR + WBC | **0.869**  (0.775–0.964) | **85.0**  (70.2–94.3) | **88.9**  (70.8–97.6) | **7.65**  (2.61–22.4) | **0.17**  (0.08–0.36) | **91.9**  (78.1–98.3) | **80.0**  (61.4–92.3) | **86.6**  (76.0–93.7) |
| CRP + ESR + IL-6 | **0.789**  (0.660–0.919) | **70.4**  (49.8–86.2) | **87.5**  (67.6–97.3) | **5.63**  (1.90–16.7) | **0.34**  (0.19–0.62) | **86.4**  (65.1–97.1) | **72.4**  (52.8–87.3) | **78.4**  (64.7–88.7) |

7. The study concludes that combining biomarkers improves diagnostic performance; however, there is limited discussion on clinical feasibility, particularly since IL‑6 testing may not be routinely available. The impact of comorbidities—e.g., infections, anemia, hepatic or renal dysfunction—on CRP, ESR, ALP, or WBC levels should be acknowledged. How these factors may confound biomarker interpretation should be discussed

Reply: Pages 22 lines 395-403 now read: Comorbid conditions in AIGA patients, including concurrent infections, anemia, hepatic diseases, renal impairment, and other inflammatory states, may influence biomarker level independently of disease activity. These factors may result in either overestimation or underestimation of biomarker performance when distinguishing active disease from remission. Nevertheless, the combined use of multiple biomarkers may improve overall diagnostic accuracy in determining disease activity by mitigating the limitations of individual markers.

8. Discussion should address clinical implementation challenges

**Reply:**

Pages 20-21, lines 365-367 now read: Although anti-IFN-γ Aabs titers are a high-performance biomarker reflecting disease status, their use may be limited in some centers due to resource constrains.

Pages 21, lines 382-384 now read: WBC counts were significantly elevated during periods of active disease, supporting their utility in monitoring disease activity in patients with anti-IFN-γ AAbs.

Pages 22, lines 394-396 now read: These results suggest that IL-6 may serve as a supplementary biomarker but is less reliable than other inflammatory biomarkers in use alone. However, its availability may be limited in routine clinical practice.

Pages 22, lines 401-403 now read: the combined use of multiple biomarkers may improve overall diagnostic accuracy in determining disease activity by mitigating the limitations of individual markers.

Pages 23, lines 411-415 now read: Although, the combination of CRP, ESR, and WBC was less accurate (AUC = 0.869), it still exhibited moderate diagnostic performance, suggesting that this simpler panel may serve as a practical and more accessible option for routine clinical monitoring, particularly in resource-limited settings.

Minor Comments
1. The table on comorbidities is only described in text. Once tables are added, ensure demographic and clinical characteristics are clearly presented.

**Reply:** Pages 10, Table 1 now read

| Characteristics | n, (%) |
| --- | --- |
| Age (years), Mean ± SD | 58.38 ± 8.55 |
| Sex |  |
| Male | 15 (38.5) |
| Female | 24 (61.5) |
| Age onset (years), Mean ± SD | 54.89 ± 9.09 |
| Active disease | 41 (59.4) |
| Thailand region of birth |  |
| Central | 18 (46.2) |
| Northeast | 13 (33.3) |
| North | 5 (12.8) |
| South | 2 (5.1) |
| West | 1 (2.6) |
| Comorbid diseases |  |
| Metabolic diseases | 24 (61.5) |
| Hypertension | 10 (26.3) |
| Dyslipidemia | 8 (20.5) |
| Diabetic mellitus | 4 (10.3) |
| Cardiovascular disease | 2 (5.1) |
| Renal disease | 7 (17.9) |
| Gastrointestinal disease | 6 (15.4) |
| Autoimmune disease | 5 (12.8) |
| Hematologic disease | 5 (12.8) |
| Neurological disease | 3 (7.7) |
| Solid malignancy | 1 (2.6) |
| Other* | 11 (28.2) |
| Clinical presentation |  |
| Lymphadenopathy | 34 (87.2) |
| Fever | 26 (66.7) |
| Reactive dermatosis | 21 (53.8) |
| Wasting | 19 (48.7) |
| Bone pain | 14 (35.9) |
| Pulmonary infection | 13 (33.3) |
| Skin infection | 7 (17.9) |
| Abdominal pain | 2 (5.1) |
| Dermatologic manifestation |  |
| Neutrophilic dermatosis | 18 (46.2) |
| Erythema nodosum | 4 (10.3) |
| Pustular | 2 (5.1) |
| Others ‡ | 1 (2.5) |
| Oppurtunistic infection † |  |
| Dissiminated nontuberculous mycobacterial infection | |
| *M.Abscessus* | 21(53.8) |
| *M.avium complex* | 8 (20.5) |
| *M.fortuitum* | 2 (5.1) |
| Other NTM species | 6 (15.4) |
| Salmonella *spp.* | 11 (28.2) |
| *Mycobacterium tubercolosis* infection | 5 (12.8) |
| Cryptococcosis | 4 (10.5) |
| Talaromycosis | 1 (2.6) |
| Others | 11 (28.2) |

2. Some thresholds (e.g., IL‑6 >16) should indicate units explicitly (pg/mL). Make units consistent throughout.

Reply: Pages 3, lines 49 now read CRP (≥ 18 mg/dL), WBC (≥ 10,455 cells/µL), and IL-6 (≥ 16 pg/mL)

3. The discussion is generally sound, but could be strengthened by comparing predictive performance with prior studies more explicitly and adding comments on how inhibitory ELISA may complement or replace indirect ELISA in clinical practice.

Reply: Pages 20-21, lines 361-367 now read: Measurement of anti-IFN-γ AAb titers by inhibitory ELISA may further enhance the diagnostic workflow by directly assessing functional neutralization of IFN-γ. This assay may complement indirect ELISA, particularly in cases with borderline titers or discordant clinical features. Although anti-IFN-γ Aabs titers are a high-performance biomarker reflecting disease status, their use may be limited in some centers due to resource constrains.

6. PLOS authors have the option to publish the peer review history of their article ([what does this mean?](https://track.editorialmanager.com/CL0/https:%2F%2Fjournals.plos.org%2Fplosone%2Fs%2Feditorial-and-peer-review-process%23loc-peer-review-history/1/010f019b68459781-aa6d89f1-b860-43fb-b1dc-94fbe767fbee-000000/dpVmzOKroLfZQTvz6VR0n261VjwSq7VvrglUcjJlpAE=243)). If published, this will include your full peer review and any attached files.


If you choose “no”, your identity will remain anonymous but your review may still be made public.


**Do you want your identity to be public for this peer review?** For information about this choice, including consent withdrawal, please see our [Privacy Policy](https://track.editorialmanager.com/CL0/https:%2F%2Fwww.plos.org%2Fprivacy-policy/1/010f019b68459781-aa6d89f1-b860-43fb-b1dc-94fbe767fbee-000000/bAacnlQ__VnzNw_CiLjD_Wt6VgRZWJxuM83z9L7chHs=243).

Reviewer #1: **Yes:** Luis Molto Delgado

Reviewer #2: No
